# Di(arylcarbazole) Substituted Oxetanes as Efficient Hole Transporting Materials with High Thermal and Morphological Stability for OLEDs

**DOI:** 10.3390/molecules28052282

**Published:** 2023-03-01

**Authors:** Daiva Tavgeniene, Baohua Zhang, Saulius Grigalevicius

**Affiliations:** 1 Department of Polymer Chemistry and Technology, Kaunas University of Technology, Radvilenu Plentas 19, LT50254 Kaunas, Lithuania; 2 Center for Advanced Analytical Science, Guangzhou Key Laboratory of Sensing Materials & Devices, School of Chemistry and Chemical Engineering, Guangzhou University, Guangzhou 510006, China

**Keywords:** carbazole, oxetane, hole transporting material, thermal stability, glass transition temperature, organic light emitting diode

## Abstract

A group of di(arylcarbazole)-substituted oxetanes has been prepared in Suzuki reactions by using the key starting material 3,3-di[3-iodocarbazol-9-yl]methyloxetane and various boronic acids (fluorophenylboronic acid, phenylboronic acid or naphthalene-1-boronic acid). Full characterization of their structure has been presented. The low molar mass compounds represent materials having high thermal stability with 5% mass loss thermal degradation temperatures in the range of 371–391 °C. Glass transition temperatures of the materials are also very high and range from 107 °C to 142 °C, which is a big advantage for formation of stable amorphous layers for optoelectronic devices, i.e., organic light emitting diodes. Hole transporting properties of the prepared materials were confirmed in formed organic light emitting diodes with tris(quinolin-8-olato)aluminium (Alq3) as a green emitter, which also served as an electron transporting layer. In the device’s materials, 3,3-di[3-phenylcarbazol-9-yl]methyloxetane (**5**) and 3,3-di[3-(1-naphthyl)carbazol-9-yl]methyloxetane (**6**) demonstrated superior hole transporting properties than that of material 3,3-di[3-(4-flourophenyl)carbazol-9-yl]methyloxetane (**4**) based device. When material 5 was used in the device structure, the OLED demonstrated rather low turn-on voltage of 3.7 V, luminous efficiency of 4.2 cd/A, power efficiency of 2.6 lm/W and maximal brightness exceeding 11670 cd/m^2^. HTL of **6** based device also showed exclusive OLED characteristics. The device was characterized by turn-on voltage of 3.4 V, maximum brightness of 13193 cd/m^2^, luminous efficiency of 3.8 cd/A and power efficiency of 2.6 lm/W. An additional hole injecting-transporting layer (HI-TL) of PEDOT considerably improved functions of the device with HTL of compound **4**. The modified OLED with a layer of the derivative **4** demonstrated exclusive characteristics with turn-on voltage of 3.9 V, high luminous efficiency of 4.7 cd/A, power efficiency of 2.6 lm/W and maximal brightness exceeding 21,000 cd/m^2^. These observations confirmed that the prepared materials have a big potential in the field of optoelectronics.

## 1. Introduction

The advantages of OLED (organic light emitting diode)-based technologies as brightness, contrast ratio, production cost, viewing angle and possibility of flexible displays are not possible by liquid crystals-based displays [1,2,3,4,5,6,7]. It is well known that multilayer structure devices having a hole transporting layer (HTL), emitting layer and electron transporting layer should be formed for efficient light emission [8,9,10,11]. One method that is very widely used to improve efficiencies of the OLED devices is the insertion of effective hole transporting layers in the construction of the OLEDs [12,13,14,15]. The positive charge transporting materials could be of low molar mass [16,17,18] or polymeric derivatives having electroactive chromophores attached to the main polymeric chains [19,20,21]. Polymeric materials usually have high thermal stability, which is an important characteristic for application in OLEDs, and form more homogenous amorphous films; however, the materials have more impurities because they cannot be purified by using chromatographic methods, which is an advantage in the case of low molar mass derivatives. On the other hand, thermal stability of the low molecular weight derivatives and stability of their amorphous films could be increased by introducing oxetane rings in structures of the mentioned derivatives. The planar structure of such derivatives minimizes the strain in the ring, and there are gauche interactions between the molecules, which increase thermal stability of the materials as well as stability of amorphous films [22,23,24].

Solution processed low molar mass derivatives containing substituted carbazole fragments are among the most studied materials for application in positive charge transporting layers due to their good chemical and environmental stability as well as high hole mobility [25,26,27]. We have reported earlier that aryl-substituted carbazoles demonstrate suitable ionization potentials and positive charge transporting properties for application in OLED devices; however, the materials had an evident tendency for crystallization [28]. Here, we report on oxetanes containing aryl-substituted carbazole fragments as electroactive chromophores. The materials demonstrated high thermal stability with high glass transition temperatures. They were tested as hole transporting layers in OLEDs with an Alq_3_ emitter, with preparation of layers for the devices by spin-coating from solutions.

## 2. Results and Discussion

**Scheme 1:**
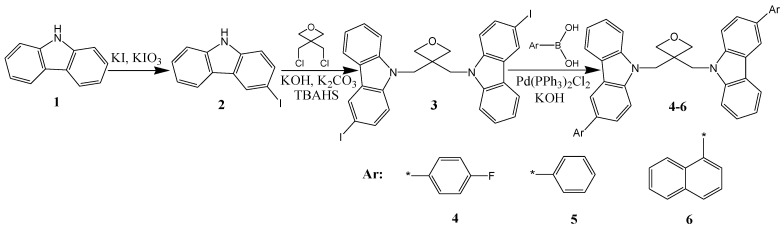
The multi-step synthetic route.

Synthesis of the twin electroactive materials **4**–**6** containing aryl-substituted carbazole rings was organized by the multi-step synthetic route as shown in Scheme 1. First, 3-Iodo-9*H*-carbazole (**2**) was prepared in well-known Tucker iodination reaction from starting material 9H-carbazole (**1**) [29]. An excess of the iodo having compound **2** was used in its reaction with 3,3-bis(chloromethyl)oxetane in order to synthesize the key starting derivative 3,3-di[3-iodocarbazol-9-yl]methyloxetane (**3**). The objective electroactive materials **4**–**6** were finally obtained from the key compound **3** by its Suzuki reactions with corresponding boronic acids, i.e., 4-fluorophenylboronic acid, phenylboronic acid or 1-naphtalene boronic acid. Structures of the newly synthesized objective derivatives **4**–**6** were characterized by mass spectrometry and nuclear magnetic resonance (NMR) spectroscopy. The spectroscopic data were found to be in good agreement with the demonstrated structures of the new materials.

**Figure 1:**
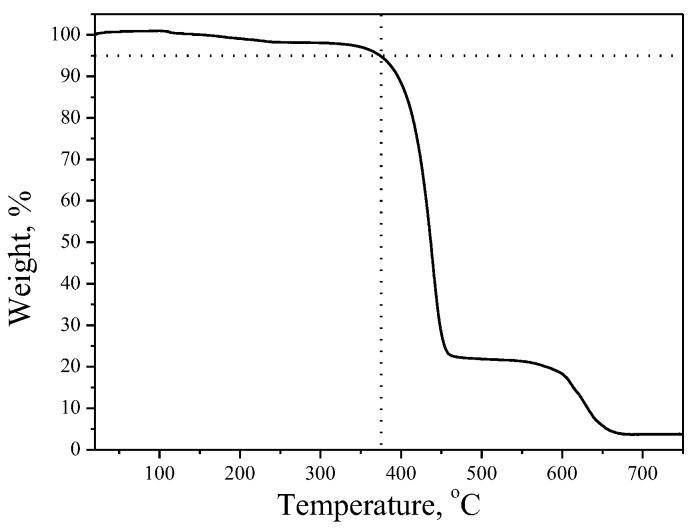
Thermo-gravimetric analysis curve of material **4**.

The behavior under heating of these materials was investigated by using DSC (differential scanning calorimetry) and TGA (thermo-gravimetric analysis) methods. The characterizations were performed under nitrogen atmosphere. It was established that all the materials are characterized by high thermal stabilities. The TGA curve of material **4** is demonstrated in Figure 1 as an example for the group. It is demonstrated that 5% mass loss was established at 378 °C for material **4**. Other objective materials also have high thermal stability with 5% mass loss at 371 °C for material **5** and at 391 °C for **6** [30], as it was obtained during the TGA test with a heating rate of 10 °C/min. The derivative **6** with naphthalene fragments demonstrates the highest thermal stability, probably due to the highest molecular weight of the compound.

**Figure 2:**
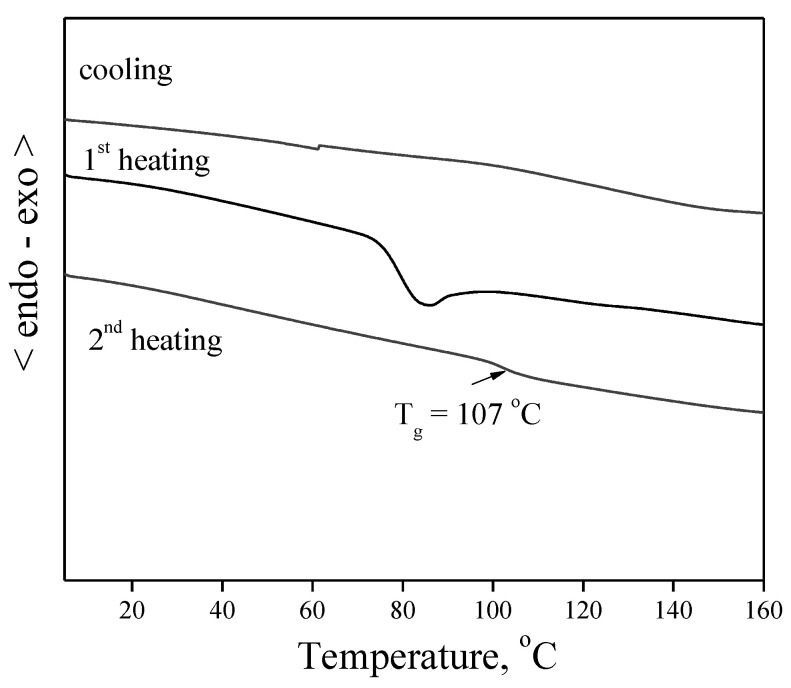
DSC curves of material **5**. Heating rate was 10 °C/min.

DSC measurements have confirmed that the synthesized derivative **5** is fully amorphous material with clearly expressed high glass transition temperature (T_g_). Results of the measurement are presented in Figure 2. The curve of the second heating of the DSC characterization, which shows the most exact T_g_ of the material, demonstrates a value of 107 °C. No peaks due to melting or crystallization were obtained during all the measurements; i.e., the material is fully amorphous and well suitable for preparation of thin homogeneous layers.

**Figure 3:**
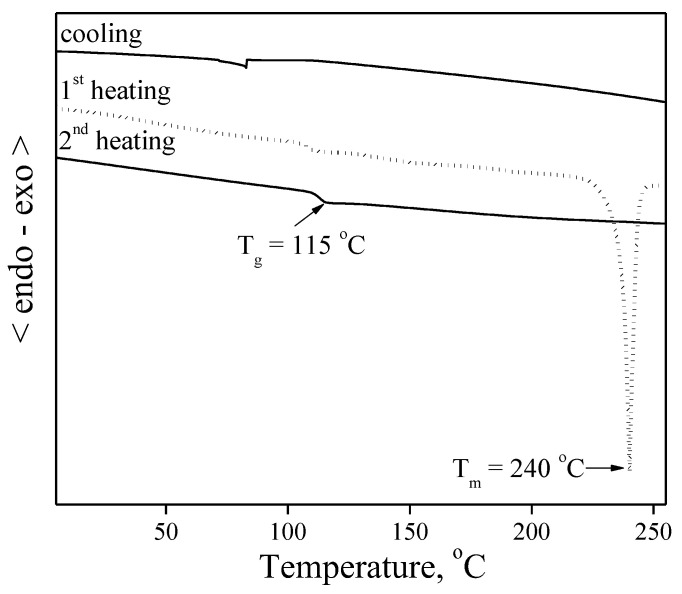
DSC curves of material **4**. Heating rate was 10 °C/min.

Other objective compounds **4** and **6** were obtained as crystalline materials after synthesis and purification by column chromatography as confirmed by the DSC; however, both of them could also form an amorphous state as it was established by the analyses. As an example for the statement, DSC curves of compound **4** are shown in Figure 3. When the crystalline sample of **4** was heated, an endothermic peak due to melting was observed at 240 °C. When the melt sample was cooled down, it formed an amorphous material. During the second heating of the sample, only glass transition temperature was registered at 115 °C, confirming the ability of the material to form amorphous films. The derivative **6** showed an analogous thermal behavior in the DSC as compound **4**. It melted at 250 °C, formed an amorphous material during cooling and demonstrated only T_g_ of 142 °C at the second heating scan [30].

It could be stated that T_g_ values of the materials depend on chemical compositions of chromophores of the twin derivatives. The naphthyl substituted materials **6** with the bulky fragments have considerably higher T_g_ than those of similar derivatives **4** and **5,** having (fluoro)phenyl substituted carbazole rings. On the other hand, T_g_ values of all the described objective materials are high, and the derivatives are well-suited for formation of amorphous layers for optoelectronic devices.

Ionization potentials (I_p_) of thin films of the materials were measured by an electron photoemission method. It was established that the I_p_ values were 5.85 eV for the layer of derivative **4**, 5.8 eV for **5** and 5.65 eV for **6**. It could be seen that ionization potentials of some materials were rather high, and an additional hole injecting–transporting layer (HI-TL) of PEDOT could be used for easier injection of holes from the ITO anode into emitting layers.

Hole transporting properties of layers of the synthesized derivatives with electroactive electron donating chromophores **4**–**6** were tested in hole transporting layers (HTL) of first generation (fluorescent) electroluminescent OLED devices, which were formed on glass substrates having an ITO (indium tin oxide) anode. It should be mentioned that all the derivatives could compose electroactive homogenous thin layers suitable for the devices by using simple spin-coating from their solutions method. A commercial emitter of Alq_3_ was used as the electroluminescent layer, which also has the function of an electron transporting component. The cathode was formed from an aluminium layer also in composition with a thin LiF electron injecting layer. The structure of the devices was ITO/HTL of **4**–**6**/Alq_3_/LiF/Al. Experimental details about formation of the devices are presented in electronic supporting information. It should be mentioned that HTL of different thicknesses (40-60-80 nm) were tested for all the synthesized materials. For comparison of hole transporting properties, an analogous device using HTL of commercial hole transporting material PEDOT (poly(3,4-ethylenedioxythiophene)-poly(styrenesulfonate)) was also constructed.

**Figure 4:**
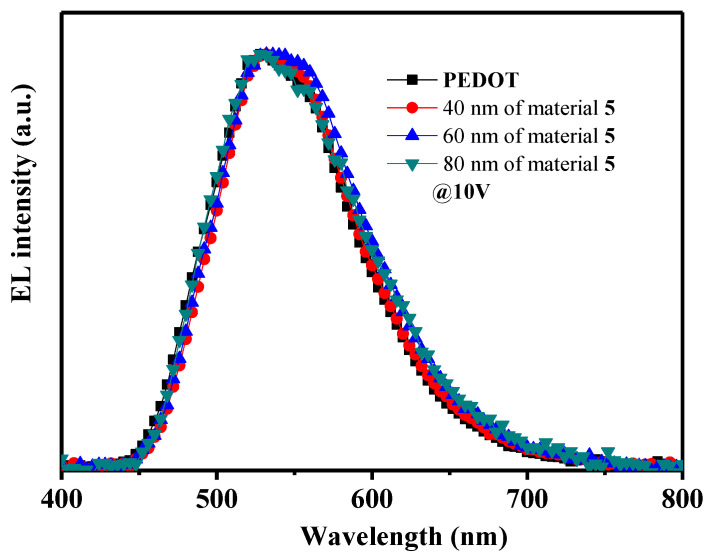
Electroluminescent spectra of the devices ITO/PEDOT or material **5** (40-60-80 nm)/Alq_3_/LiF/Al at voltage of 10 V.

When the formed OLED devices were connected to forward bias, bright green electroluminescence (EL) originating from the emitting layer of Alq_3_ was observed with an emission maximum at around 520 nm for all the devices. As an example, Figure 4 demonstrates the EL spectra of devices using derivative **5** as HTL of different thicknesses (40-60-80 nm). The EL spectrum of OLED using PEDOT as HI-TL is also shown in the Figure for comparison. These measurements confirmed that the synthesized materials **4**–**6** only served as hole transporting layers without any exciplex formation at the junction with the Alq_3_ emitting layer. The data also demonstrate that hole injection and mobility in the thin hole transporting films of the compounds **4**–**6** were fully sufficient for an effective charge carrier’s recombination occurring within the Alq_3_ emitter layer.

The current density–voltage (I–V) curves of the described devices are depicted in electronic supporting information (ESI) in Figure S1 and demonstrate typical OLED behavior. The luminance–voltage and current efficiency–luminance characteristics of the devices are also presented in Figures S2 and S3 of ESI. The rather low turn-on voltages of 3.4–4.1 V were demonstrated by the luminance–voltage function for some devices having suitable thickness of HTL. All the OLED characteristics, including turn-on voltages (V_on_), maximum brightness (L_max_), maximum luminous efficiency (LE) as well as power efficiency (PE), are summarized in Table 1. The characteristics of a device with PEDOT as HI-TL material are also presented in the Table for comparison of the properties.

It is evident that OLEDs using HTL materials **5** and **6** demonstrated similar and better OLED characteristics as compared with those of devices using HTL of derivative **4**. The rather high turn-on voltages (V_on_) of material **4** based devices using various thicknesses of the layers could confirm that there are inappropriate hole injecting properties of positive charges from ITO anode into HTL of **4**. On the other hand, materials **5** and **6** demonstrated superior OLED characteristics to those of the material **4** based device. When 40 nm layer of material **5** was used in an analogous device structure, the OLED demonstrated rather low turn-on voltage of 3.7 V, luminous efficiency of 4.2 cd/A, power efficiency of 2.6 lm/W and maximal brightness exceeding 11,670 cd/m^2^. The HTL material **6** based device also showed exclusive OLED characteristics. In addition, 40 nm HTL using devices could be characterized by turn-on voltage of 3.4 V, maximum brightness of 13,193 cd/m^2^_,_ luminous efficiency of 3.8 cd/A and power efficiency of 2.6 lm/W. It can be seen from Table 1 that these characteristics are promising as compared to those of analogous devices using a commercial HI-TL layer of PEDOT, and the materials **5** and **6** are well suited for application in HTL.

**Figure 5:**
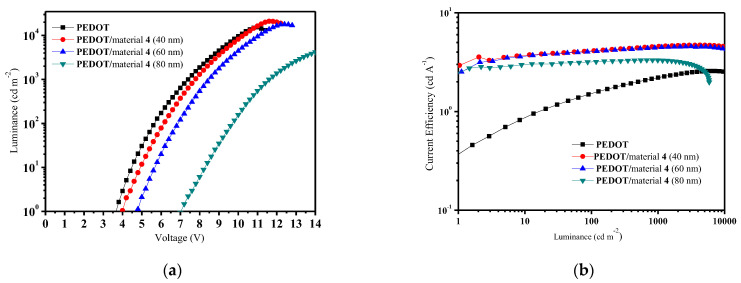
Luminance–voltage (L–V) curves (**a**) and current efficiency–luminance (CE–L) curves (**b**) of the devices with the configuration: ITO/**PEDOT**/HTL of **4** (40-60-80 nm)/Alq_3_/LiF/Al.

In order to further improve characteristics of the OLEDs, devices of structure ITO/PEDOT/HTL of material **4**, **5** or **6** (40-60-80 nm)/Alq_3_/LiF/Al using 40 nm of additional HI-TL of PEDOT were formed and characterized. It was observed that the additional layer of PEDOT did not have a noticeable influence for characteristics of devices using HTLs of derivatives **5** and **6**. However, the layer considerably improved functions of the device with HTL of compound **4**. The luminance–voltage and current efficiency–luminance characteristics of the devices ITO/PEDOT/HTL of **4** (40-60-80 nm)/Alq_3_/LiF/Al are presented in Figure 5. The modified OLED using 40 nm layer of derivative **4** demonstrated exclusive characteristics with turn-on voltage of 3.9 V, high luminous efficiency of 4.7 cd/A, power efficiency of 2.6 lm/W and maximal brightness exceeding 21,000 cd/m^2^. This observation confirmed that the additional HI-TL in combination with HTL of compound **4** could strongly improve characteristics of the described devices. In the general case, efficiencies of the described OLEDs are rather high. Earlier, we had investigated the very widely used commercial hole transporting material *N*,*N*′-bis(naphthalen-1-yl)-*N*,*N*′-bis(phenyl)benzidine (**NPB**). It was used as the HTL material in analogous devices, which are described in the paper. Maximal luminous efficiency of the OLED using **NPB** reached only 2.7 cd/A [31]. It should also be pointed out that these OLED data were measured in non-optimized test devices. The efficiencies may be further improved by an optimization of device formation conditions and by encapsulation of the OLEDs [32].

In conclusion, new electro-active materials for hole transporting layers (HTL) were synthesized, characterized and tested in OLED devices. The amorphous materials demonstrated very high thermal stability (371–391 °C) and high glass transition temperatures in a range of 107–142 °C due to incorporation of oxetane rings in their structures. In simple devices with tris(quinolin-8-olato)aluminium (Alq_3_) as an emitter as well as electron transporting layer materials 3,3-di[3-phenylcarbazol-9-yl]methyloxetane (**5**) and 3,3-di[3-(1-naphthyl)carbazol-9-yl]methyloxetane (**6**) demonstrated superior hole transporting properties than those of material 3,3-di[3-(4-flourophenyl)carbazol-9-yl]methyloxetane (**4**) based device. When the layer of material **5** was used in the device structure, the OLED demonstrated rather low turn-on voltage of 3.7 V, luminous efficiency of 4.2 cd/A, power efficiency of 2.6 lm/W and maximal brightness exceeding 11,670 cd/m^2^. The HTL of **6** based device also showed exclusive OLED characteristics. The device was characterized by turn-on voltage of 3.4 V, maximum brightness of 13,193 cd/m^2^_,_ luminous efficiency of 3.8 cd/A and power efficiency of 2.6 lm/W. An additional hole injecting–transporting layer of PEDOT considerably improved functions of the device with the HTL of compound **4**. The modified OLED using layer of derivative **4** demonstrated exclusive characteristics with turn-on voltage of 3.9 V, high luminous efficiency of 4.7 cd/A, power efficiency of 2.6 lm/W and maximal brightness exceeding 21,000 cd/m^2^. These observations confirmed that the tested HTL materials have big potential in the field of optoelectronics.

## 3. Materials

9*H*-Carbazole (**1**), 3,3-bis(chloromethyl)oxetane, 4-fluorophenylboronic acid, phenylboronic acid, naphthalene-1-boronic acid, bis(triphenylphosphine)palladium(II) dichloride (Pd(PPh_3_)_2_Cl_2_), K_2_CO_3_, KOH, KI, KIO_3_, tetra-*n*-butylammonium hydrogen sulfate (TBAHS) and Na_2_SO_4_ were purchased from Merck and used as received.

In addition, 3-Iodo-9*H*-carbazole (**2**) was synthesized according to the procedure described in the literature [29].

Preparation of 3,3-Di[3-iodocarbazol-9-yl]methyloxetane (**3**) was performed according to the same method, which we have described earlier [33].

Then, 3,3-Di[3-(4-flourophenyl)carbazol-9-yl]methyloxetane (**4**), 0.4 g (0.6 mmol) of 3,3-di [3-iodocarbazol-9-yl]methyloxetane (**3**), 0.21 g (1.5 mmol) of 4-fluorophenylboronic acid, 0.02 g (0.03 mmol) of PdCl_2_(PPh_3_)_2_ and 0.17 g (3.0 mmol) of powdered KOH were stirred in 8 mL of THF containing degassed water (1 mL) at 80 °C under nitrogen for 24 h. After TLC control, the reaction mixture was cooled and quenched by the addition of ice water. The product was extracted with chloroform. The combined extract was dried over anhydrous Na_2_SO_4_. The crude product was purified by silica gel column chromatography using the mixture of ethyl acetate and hexane (vol. ratio 1:7) as an eluent. The following resulted: yield: 0.2 g (56%) of white crystals; m.p.: 240 °C (DSC); MS (APCI^+^, 20 V): 605.68 ([M + H], 100%); ^1^H NMR (400 MHz, CDCl_3_, δ): 8.20 (s, 2H, Ar), 8.09 (d, 2H, *J* = 7.6 Hz, Ar), 7.64–7.50 (m, 6H, Ar), 7.33–7.20 (m, 6H, Ar), 7.43–7.33 (m, 2H, Ar), 7.14–6.98 (m, 4H, Ar), 4.65 (s, 4H, 2 × OCH_2_), 4.61 (s, 4H, 2 × NCH_2_); ^13^C NMR (100 MHz, CDCl_3_, δ): 141.87, 140.81, 137.88, 132.43, 128.81, 128.73, 126.44, 125.50, 123.80, 123.38, 120.73, 120.00, 119.04, 115.75, 115.54, 108.99, 108.99, 108.87, 75.95, 50.77, 47.47; FT-IR (KBr), cm^−1^: 3051, 2957, 2928, 2884, 1888, 1776, 1728, 1626, 1600, 1516, 1479, 1463, 1386, 1339, 1299, 1219, 1156, 973, 893, 837, 799, 770, 748.

Additionally, 3,3-Di(3-phenylcarbazol-9-yl)methyloxetane (**5**). 0.4 g (0.6 mmol) of 3,3-di [3-iodocarbazol-9-yl]methyloxetane (**3**), 0.18 g (1.5 mmol) of phenylboronic acid, 0.02 g (0.03 mmol) of PdCl_2_(PPh_3_)_2_ and 0.17 g (3.0 mmol) of powdered KOH were stirred in 8 mL of THF containing degassed water (1 mL) at 80 °C under nitrogen for 2 h. After TLC control, the reaction mixture was cooled and quenched by the addition of ice water. The product was extracted with chloroform. The combined extract was dried over anhydrous Na_2_SO_4_. The crude product was purified by silica gel column chromatography using the mixture of ethyl acetate and hexane (vol. ratio 1:7) as an eluent. The following resulted: yield: 0.2 g (59%) of white amorphous product; MS (APCI^+^, 20 V): 569.42 ([M + H], 100%); ^1^H NMR (400 MHz, CDCl_3_, δ): 8.27 (s, 2H, Ar), 8.10 (d, 2H, *J* = 7.6 Hz, Ar), 7.68–7.57 (m, 6H, Ar), 7.46–7.35 (m, 6H, Ar), 7.33–7.19 (m, 8H, Ar), 4.67 (s, 4H, 2 × OCH_2_), 4.62 (s, 4H, 2 × NCH_2_); ^13^C NMR (100 MHz, CDCl_3_, δ): 141.87, 141.75, 140.90, 133.41, 128.83, 127.32, 126.68, 126.35, 125.67, 123.87, 123.48, 120.72, 119.94, 119.16, 108.96, 108.85, 75.98, 50.78, 47.51; FT-IR (KBr), cm^−1^: 3051, 3024, 2950, 2925, 2866, 1874, 1626, 1600, 1533, 1476, 1460, 1384, 1334, 1297, 1229, 1156, 968, 886, 810, 761, 746, 729, 697.

Finally, 3,3-Di [3-(1-naphthyl)carbazol-9-yl]methyloxetane (**6**) was synthesized according to the same procedure, which we have described earlier [30]: m.p.: 250 °C (DSC).

## Figures and Tables

**Table 1:** OLED characteristics of the devices with the configuration: ITO/HTL of **4**–**6**/Alq_3_/LiF/Al having different thicknesses of HTLs.

	HTLs	V_on_ [V]	L_max_ [cd/m^2^]	LE [cd/A]	PE [lm/W]
**4**	40 nm	4.1	6426	3.8	2.3
	60 nm	4.9	6609	3.7	1.9
	80 nm	6.7	6239	3.7	1.4
	PEDOT	3.8	15,035	2.4	0.8
**5**	40 nm	3.7	11,672	4.2	2.6
	60 nm	4.3	10,203	4.1	2.2
	80 nm	5.7	5230	3.6	1.9
	PEDOT	3.6	14,040	2.3	0.8
**6**	40 nm	3.4	13,193	3.8	2.6
	60 nm	3.7	12,819	3.7	2.3
	80 nm	4.7	4663	3.3	1.8
	PEDOT	3.7	13,370	2.1	0.7

## Data Availability

The data presented in this study are available in supplementary material.

## Supplementary Materials

The following supporting information can be downloaded at: https://www.mdpi.com/article/10.3390/molecules28052282/s1, Figure S1. Current density–voltage (I–V) curves of the devices using HTLs of 4 (**a**), **5** (**b**) and **6** (**c**); Figure S2. Luminance–voltage (L–V) curves of the devices using HTLs of **4** (**a**), **5** (**b**) and **6** (**c**); Figure S3. Current efficiency–luminance (CE–L) curves of the devices using HTLs of **4** (**a**), **5** (**b**) and **6** (**c**); Figure S4. Thermo-gravimetric analysis curve of the material **5**; Figure S5. Thermo-gravimetric analysis curve of the material **6**; Figure S6. DSC curves of material **6**; Figure S7. Mass spectrum of material **4**; Figure S8. Mass spectrum of material **5**; Figure S9. Mass spectrum of material **6**; Figure S10. FT-IR spectrum of material **4**; Figure S11. FT-IR spectrum of material **5**; Figure S12. FT-IR spectrum of material **6**; Figure S13. 1H NMR of material **4**; Figure S14. 1H NMR of material **5**; Figure S15. 1H NMR of material **5**; Figure S16. 13C NMR of material **4**; Figure S17. 13C NMR of material **5**; Figure S18. 13C NMR of material **5**.

## Sample Availability

Samples of the compounds **4**–**6** are available from the authors.
