# Supplementary material for: Di(arylcarbazole) Substituted Oxetanes as Efficient Hole Transporting Materials with High Thermal and Morphological Stability for OLEDs
Source: Molecules. 2023 Mar 1;28(5):2282. doi: 10.3390/molecules28052282 (PMC10005742; doi:10.3390/molecules28052282)
Supplement: Supplementary file 1 [file molecules-28-02282-s001.zip › molecules-2199412-supplementary.pdf]

Electronic supporting information

Di(arylcarbazole) Substituted Oxetanes as Efficient Hole Transporting Materials with High Thermal and Morphological Stability for OLEDs

Daiva Tavgeniene [1,*], Baohua Zhang [2] and Saulius Grigalevicius [1,*]

[1] Department of Polymer Chemistry and Technology, Kaunas University of Technology, Radvilenu Plentas 19, LT50254 Kaunas, Lithuania
[2] Center for Advanced Analytical Science, Guangzhou Key Laboratory of Sensing Materials & Devices, School of Chemistry and Chemical Engineering, Guangzhou University, Guangzhou 510006, China
* Correspondence: daiva.tavgeniene@ktu.lt (D.T.); saulius.grigalevicius@ktu.lt (S.G.)

Instrumentation

^{1}H nuclear magnetic resonance (NMR) spectra were obtained using a Bruker Avance III (400 MHz) apparatus. The data are given as chemical shifts (δ) in ppm against trimethylsilane (in parenthesis: multiplicity, integration, coupling constant). Mass spectra were obtained on a Waters ZQ 2000 mass spectrometer (electron spray ionization). Infrared (IR) spectra are measured using a Vertex 70 Bruker spectrophotometer.

Differential scanning calorimetry (DSC) measurements were carried out using a Bruker Reflex II thermosystem. Thermogravimetric analysis (TGA) was performed on a TGAQ50 aparatus. The TGA and DSC curves were recorded in a nitrogen atmosphere at a heating rate of 10° C/min.

Fabrication of OLED devices

The electroluminescent OLED devices were fabricated on glass substrates. The electro-active organic layers were sandwiched between a bottom indium tin oxide (ITO) anode and a top metal cathode, and had the structure: ITO/HTLs(40-80 nm)/Alq_3(100nm)/LiF(1nm)/Al(100nm). The ITO-coated substrates were carefully cleaned and treated with UV/ozone right before deposition of the organic layers. Hole transporting (HTL) layers were prepared by spin-coating of 40, 60 or 80 nm layer of the corresponding material **4-6** onto the substrates from chloroform solutions (3-10 mg/ml). Evaporation of the emitting/electron-transporting tris(quinolin-8-olato)aluminium (Alq_3) layer (100 nm) and a LiF/Al electrode (1/100 nm) was done at a pressure of 4×10^{-4} Pa in vacuum evaporation equipment. The luminance of the fabricated devices was measured using a Minolta CS-100 luminance-meter. A Keithley 2400 electrometer was used to measure the current-voltage characteristics of the devices. All the measurements were performed at ambient conditions in air. For comparison of the properties an analogous device with 40 nm thick PEDOT:PSS (Baytron P 4083) hole injecting-transporting layer (HI-TL) was prepared and had the structure : ITO/ PEDOT:PSS (40nm)/HTLs(40-80 nm)/Alq_3(100nm)/LiF(1nm)/Al(100nm). Thickness of the layers was measured by using Alpha-Step D-500 mechanical profilometer.

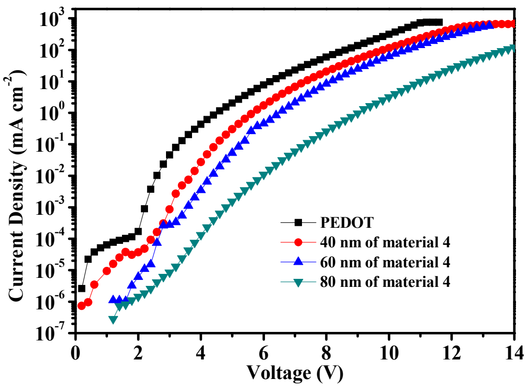

(a)

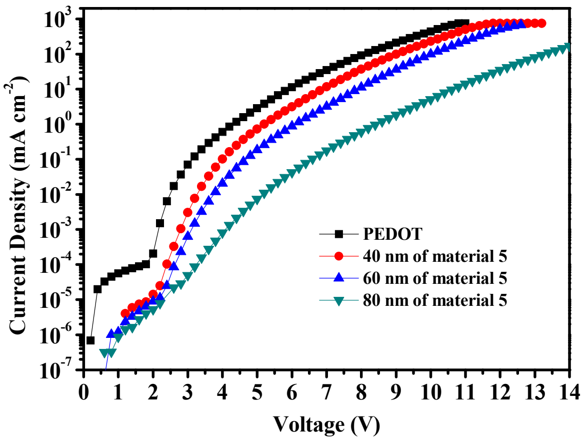

(b)

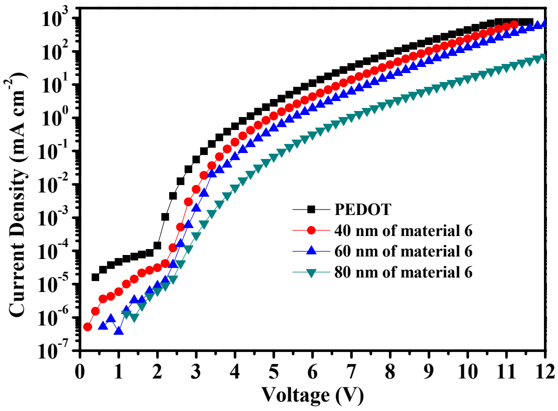

(c)

Figure S1. Current density–voltage (I–V) curves of the devices using HTLs of **4**(a), **5**(b) and **6**(c).

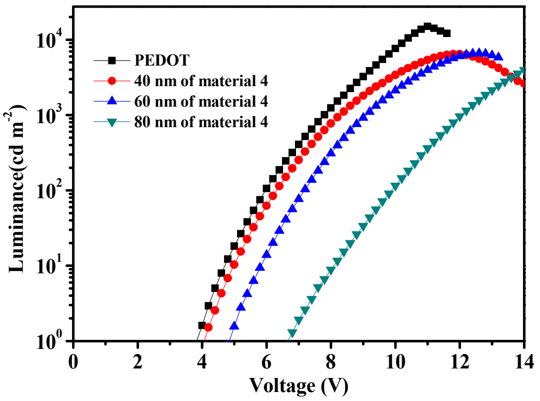

(a)

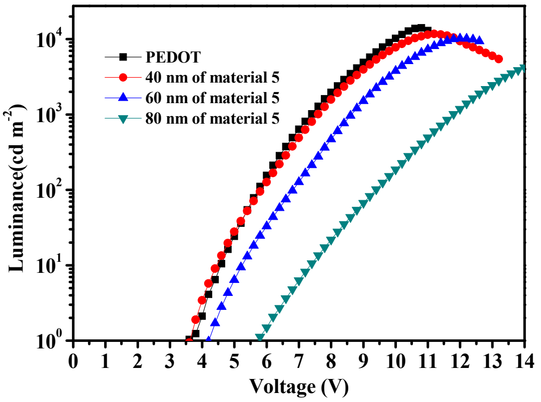

(b)

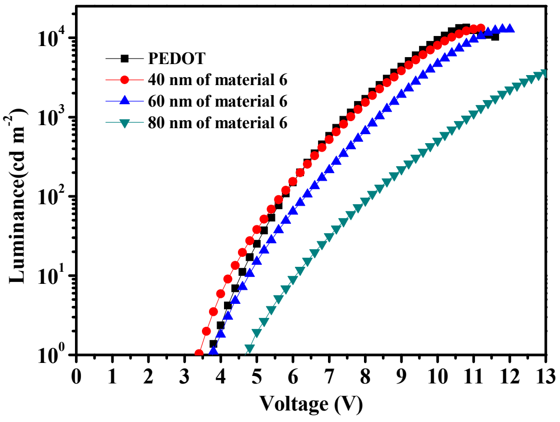

(c)

Figure S2. Luminance – voltage (L–V) curves of the devices using HTLs of **4**(a), **5**(b) and **6**(c).

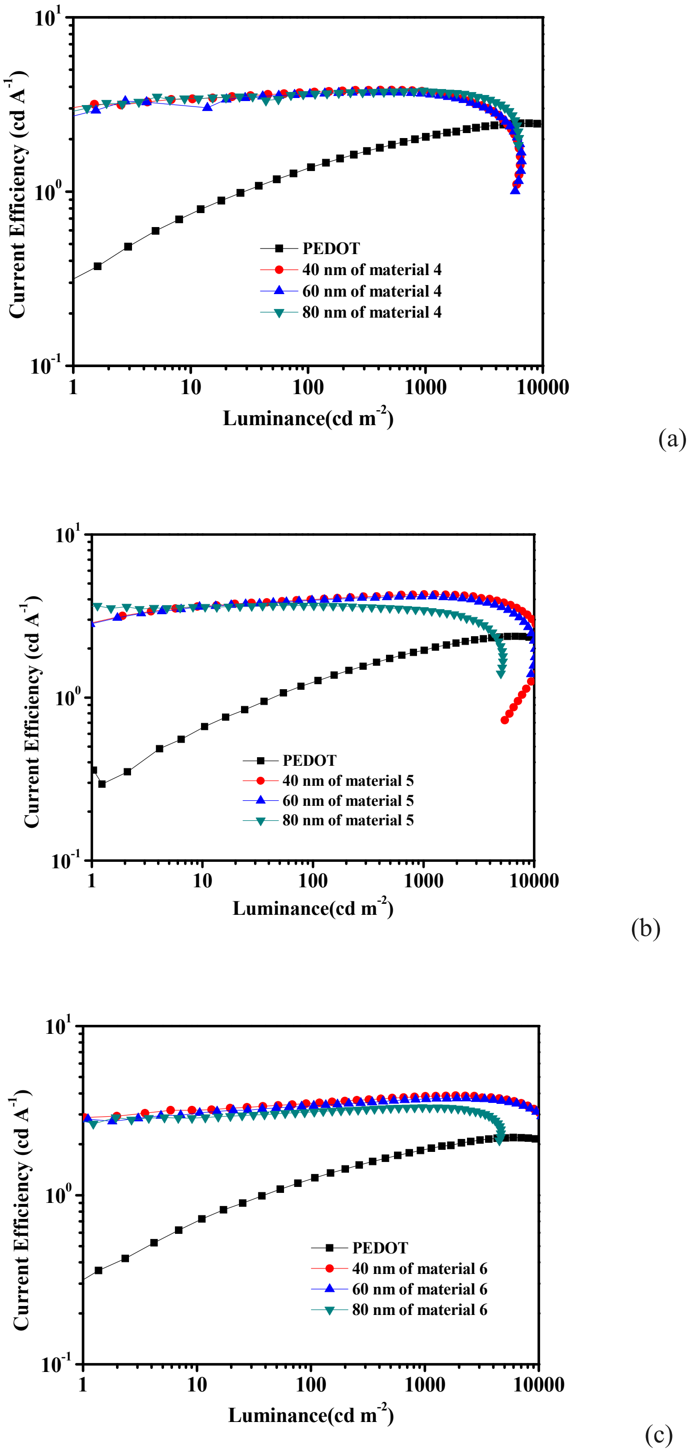

Figure S3. Current efficiency – luminance (CE–L) curves of the devices using HTLs of **4**(a), **5**(b) and **6**(c).

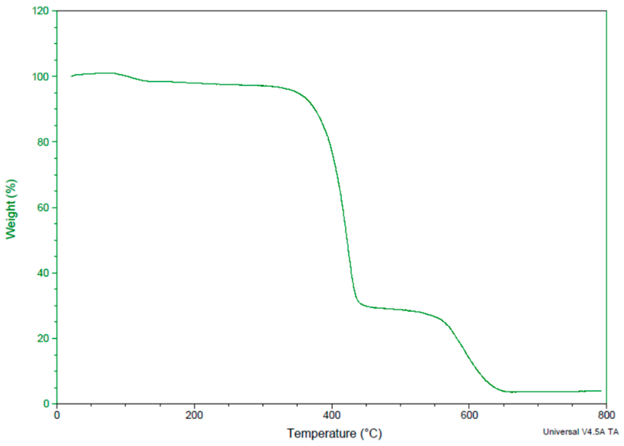

Figure S4. Thermo-gravimetric analysis curve of the material **5**.

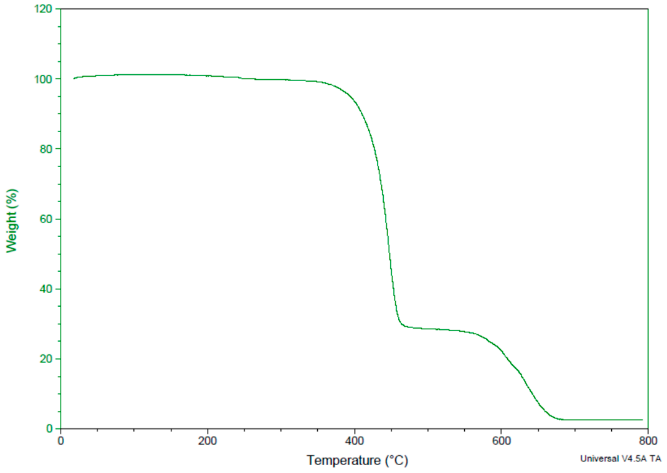

Figure S5. Thermo-gravimetric analysis curve of the material **6**.

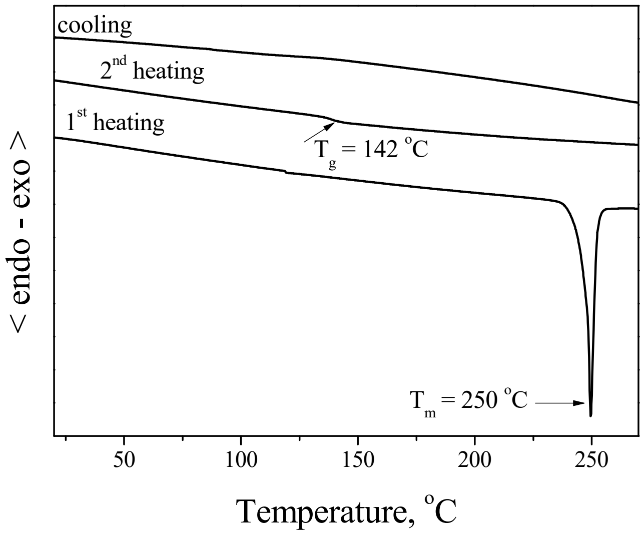

Figure S6. DSC curves of material **6**.

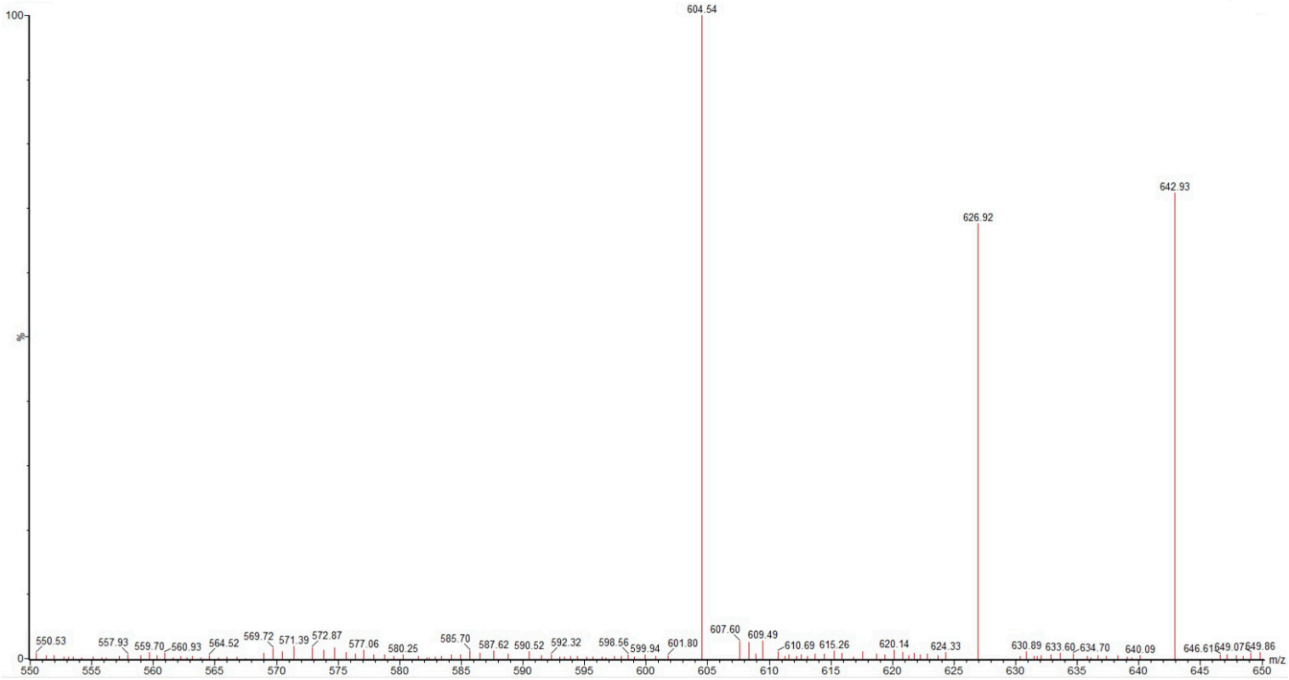

Figure S7. Mass spectrum of material **4.**

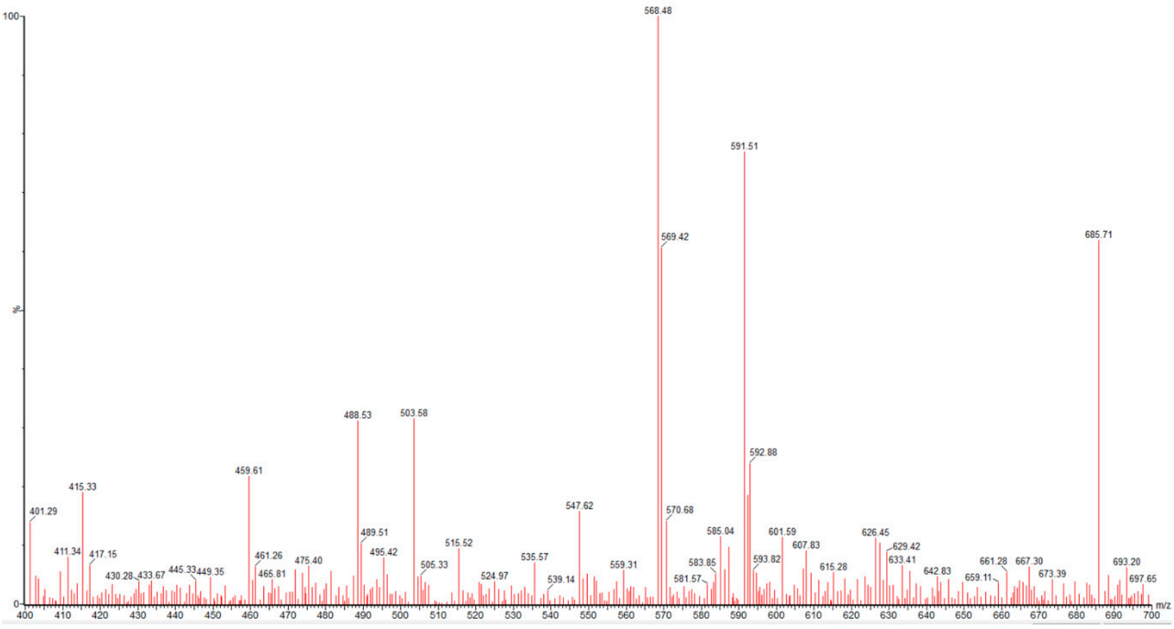

Figure S8. Mass spectrum of material **5.**

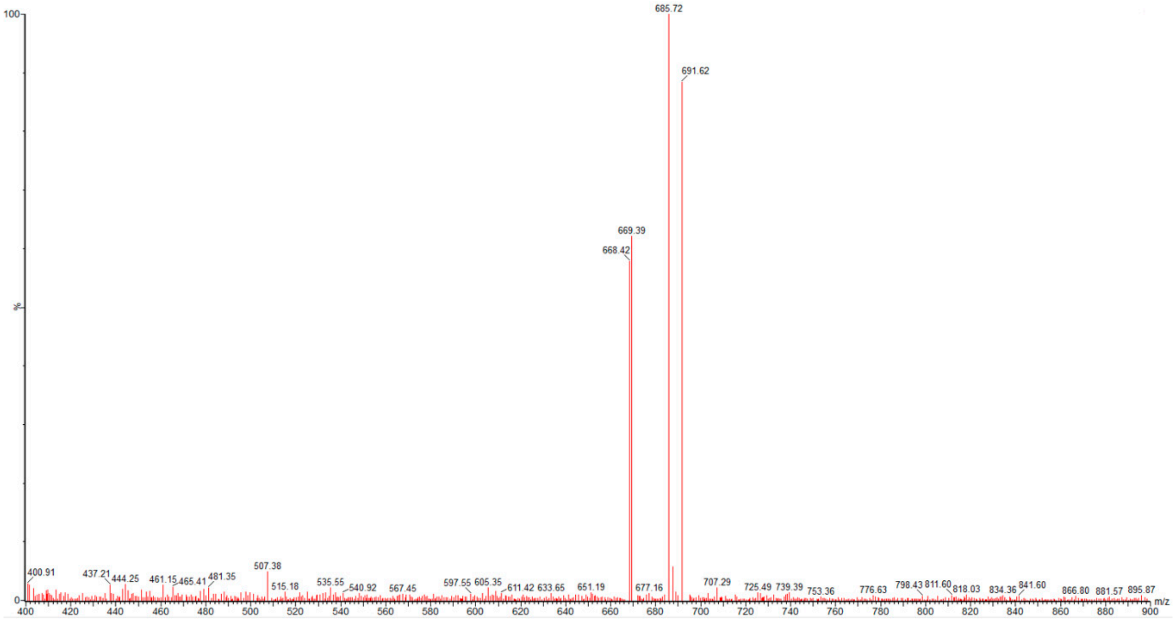

Figure S9. Mass spectrum of material **6.**

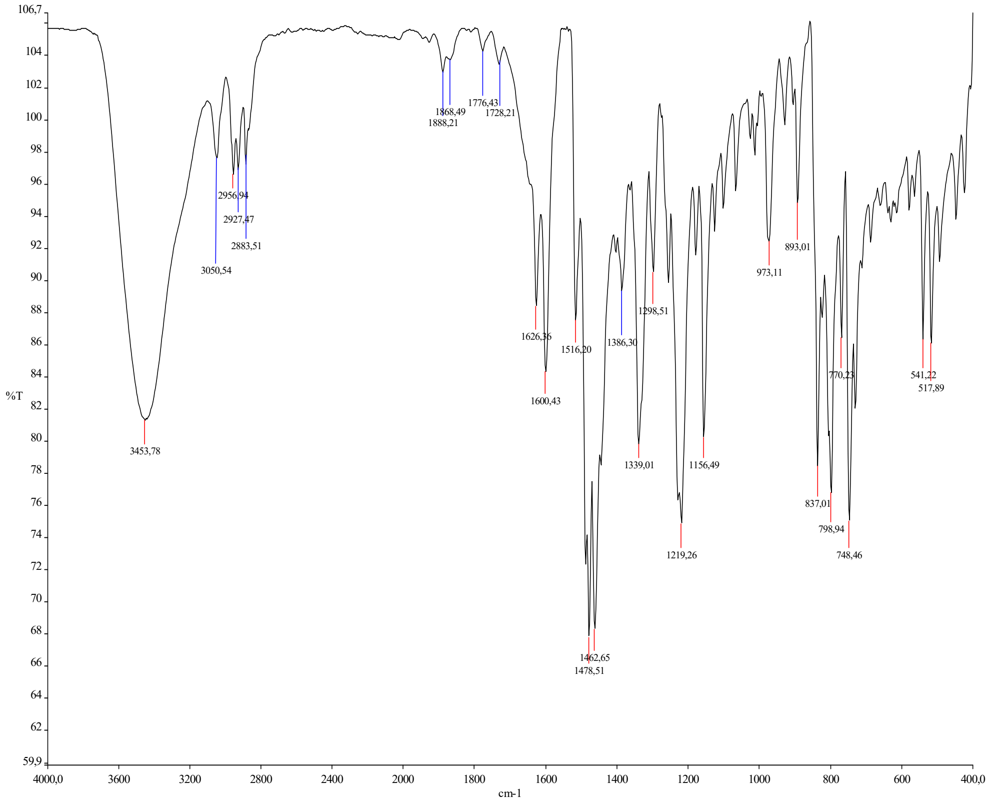

Figure S10. FT-IR spectrum of material **4.**

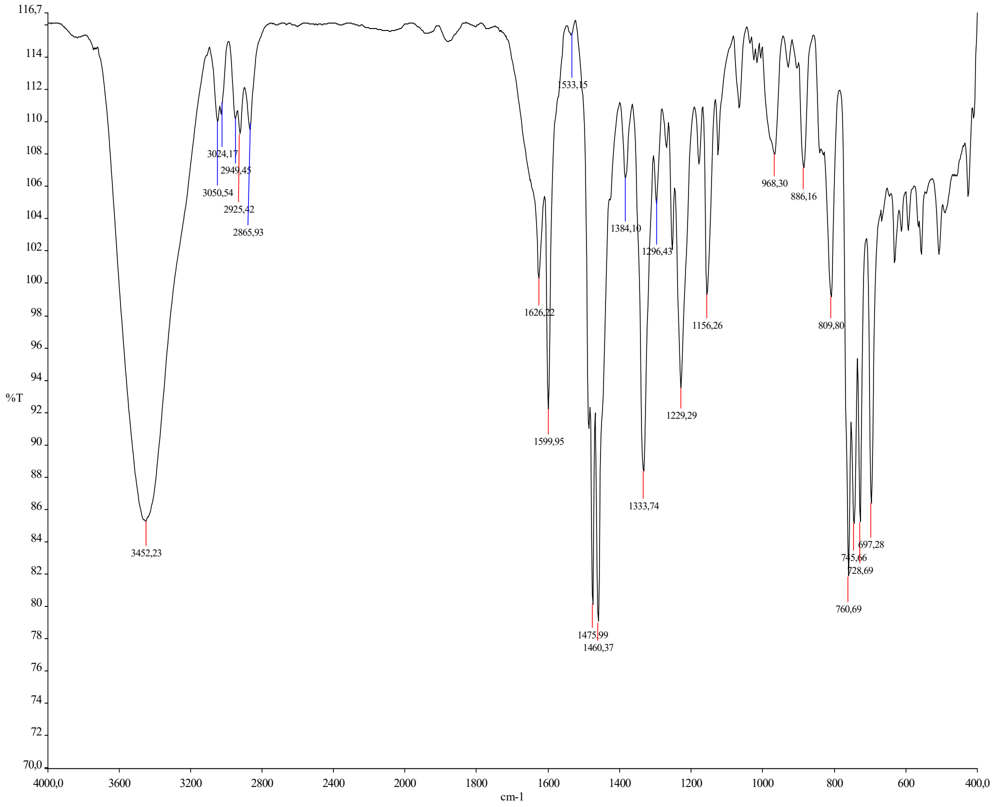

Figure S11. FT-IR spectrum of material **5.**

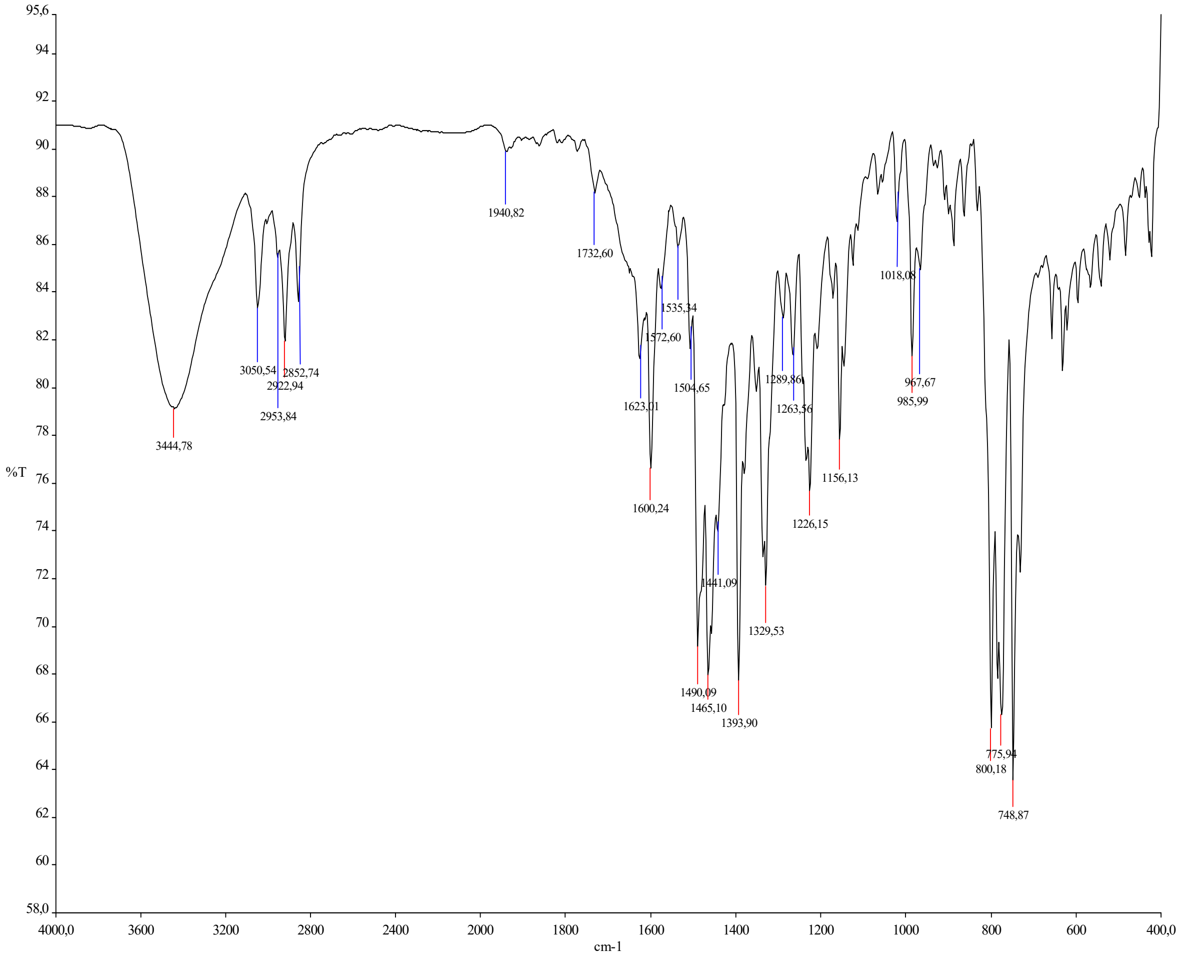

Figure S12. FT-IR spectrum of material **6.**

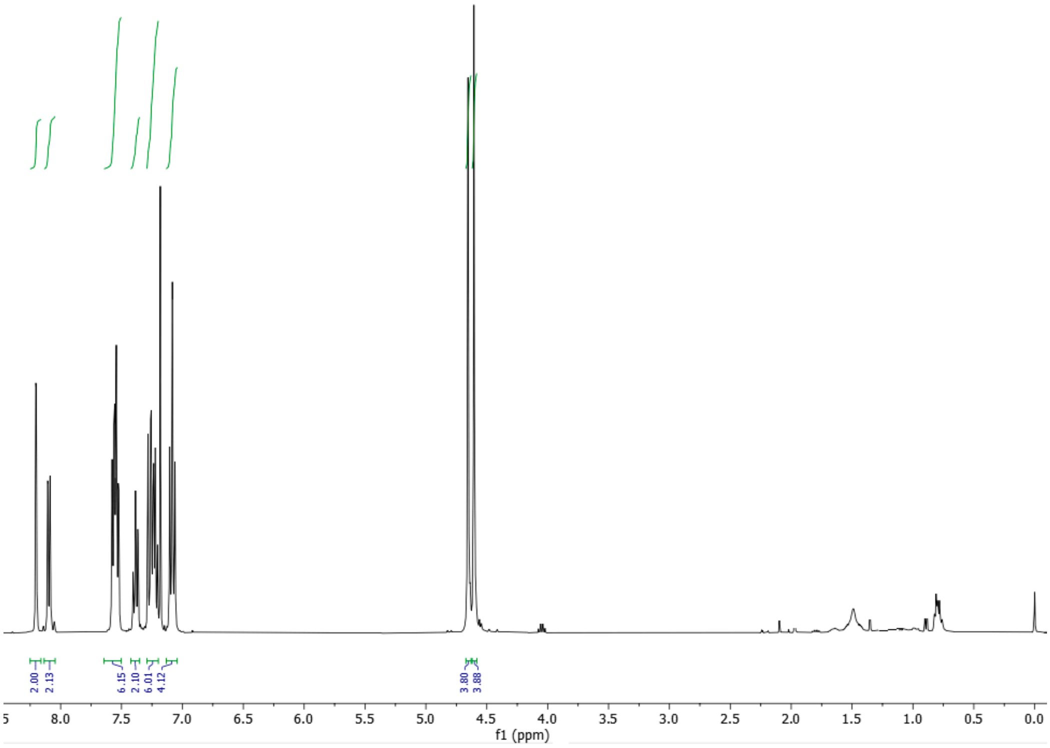

Figure S13. ^{1}H NMR of material **4.**

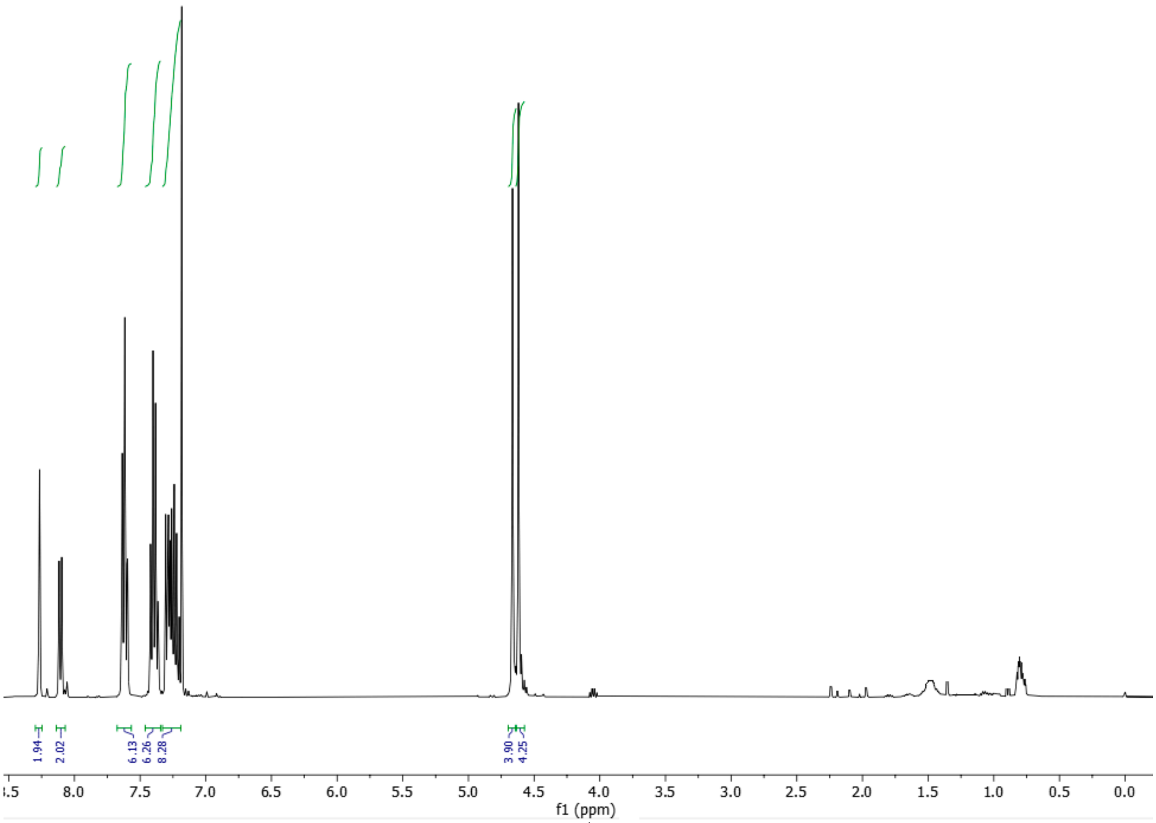

Figure S14. ^{1}H NMR of material **5.**

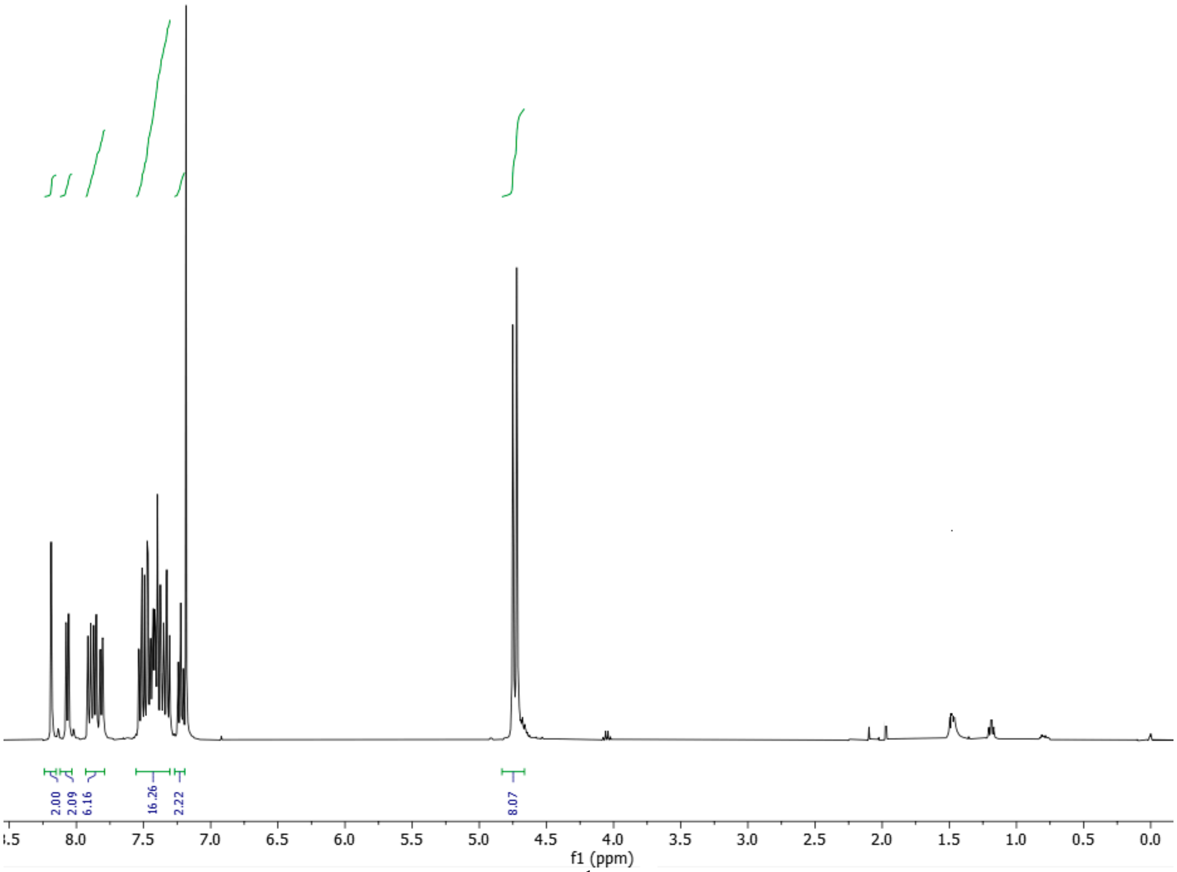

Figure S15. ^{1}H NMR of material **5.**

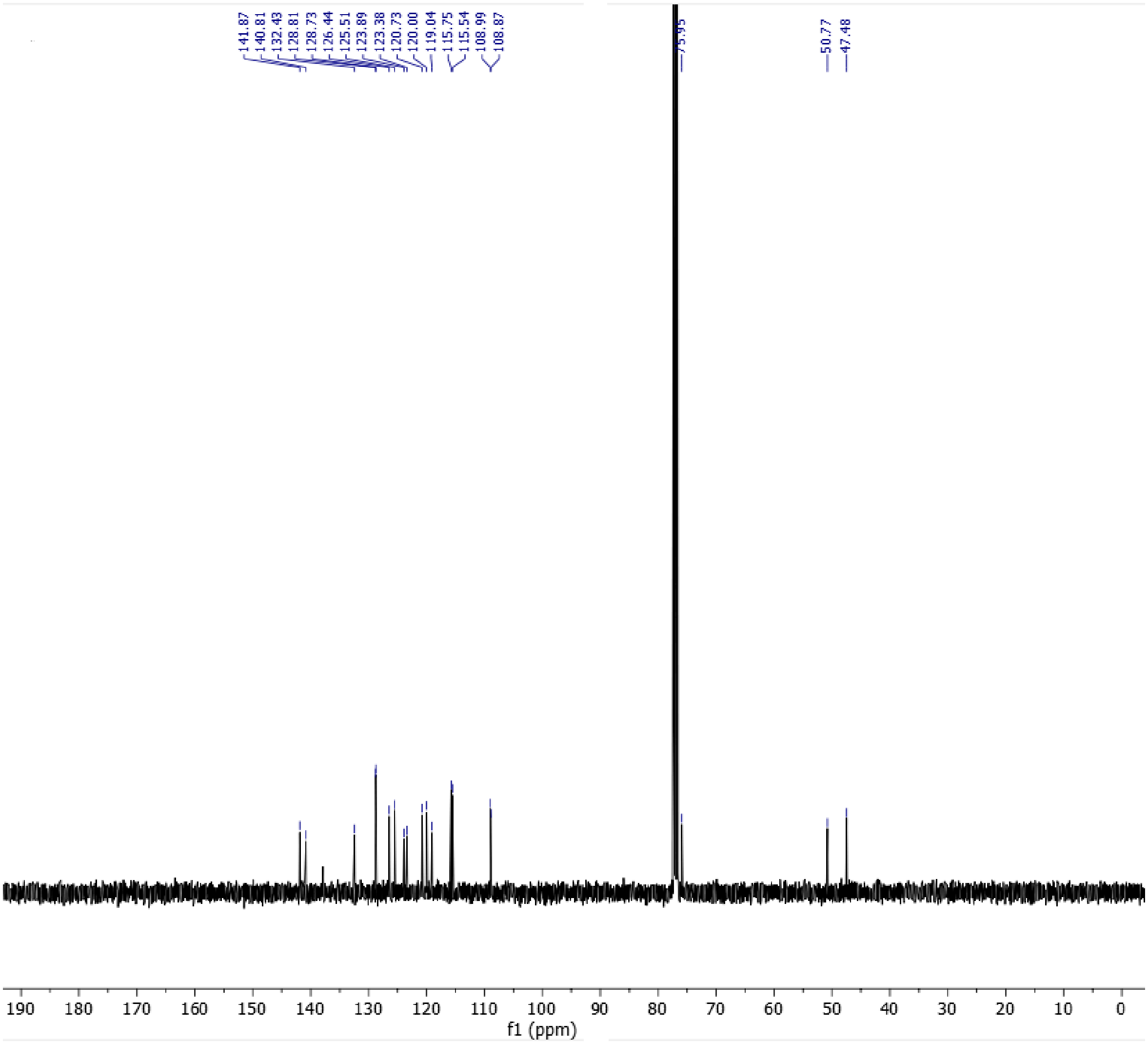

Figure S16. ^{13}C NMR of material **4.**

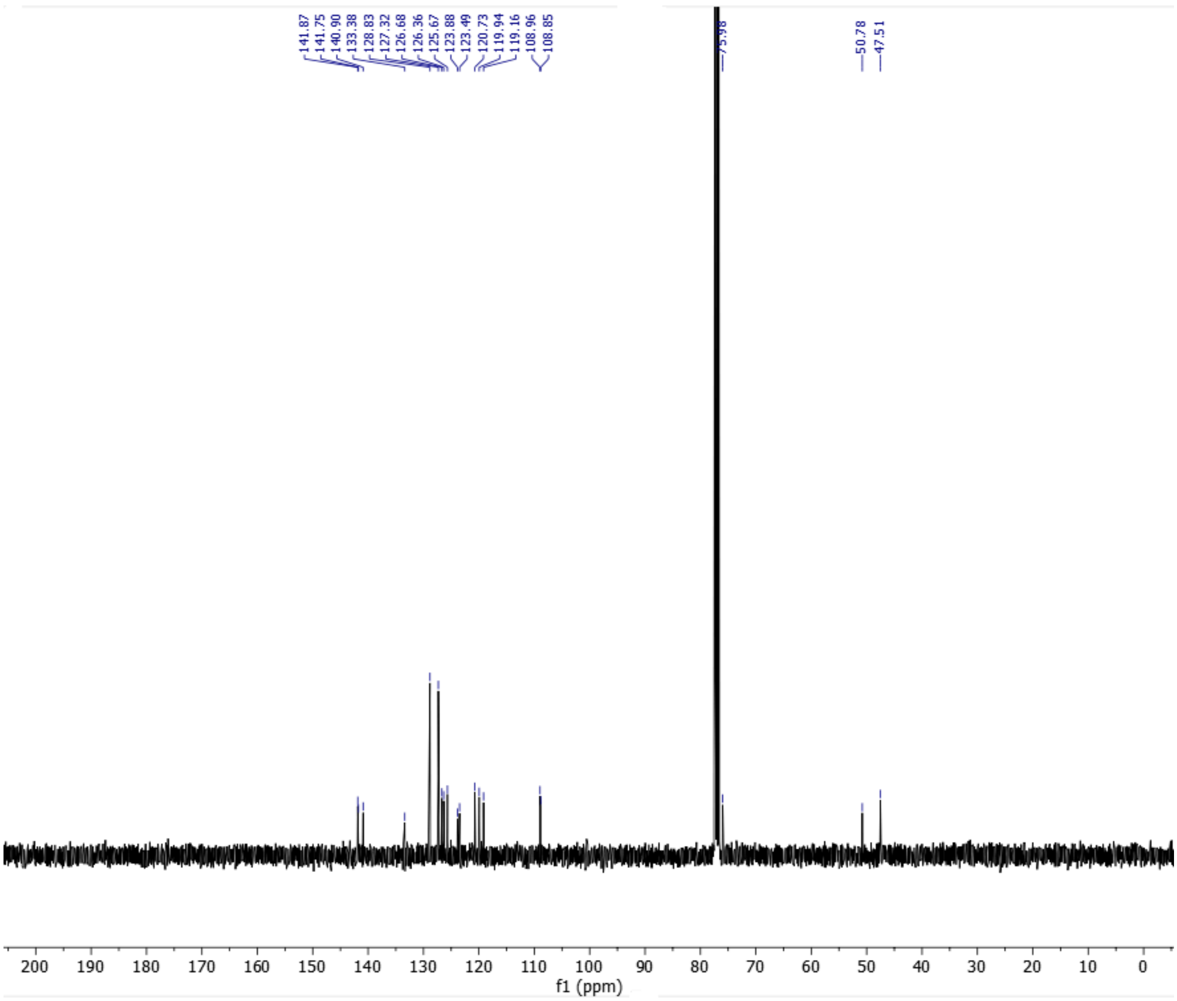

Figure S17. ^{13}C NMR of material **5.**

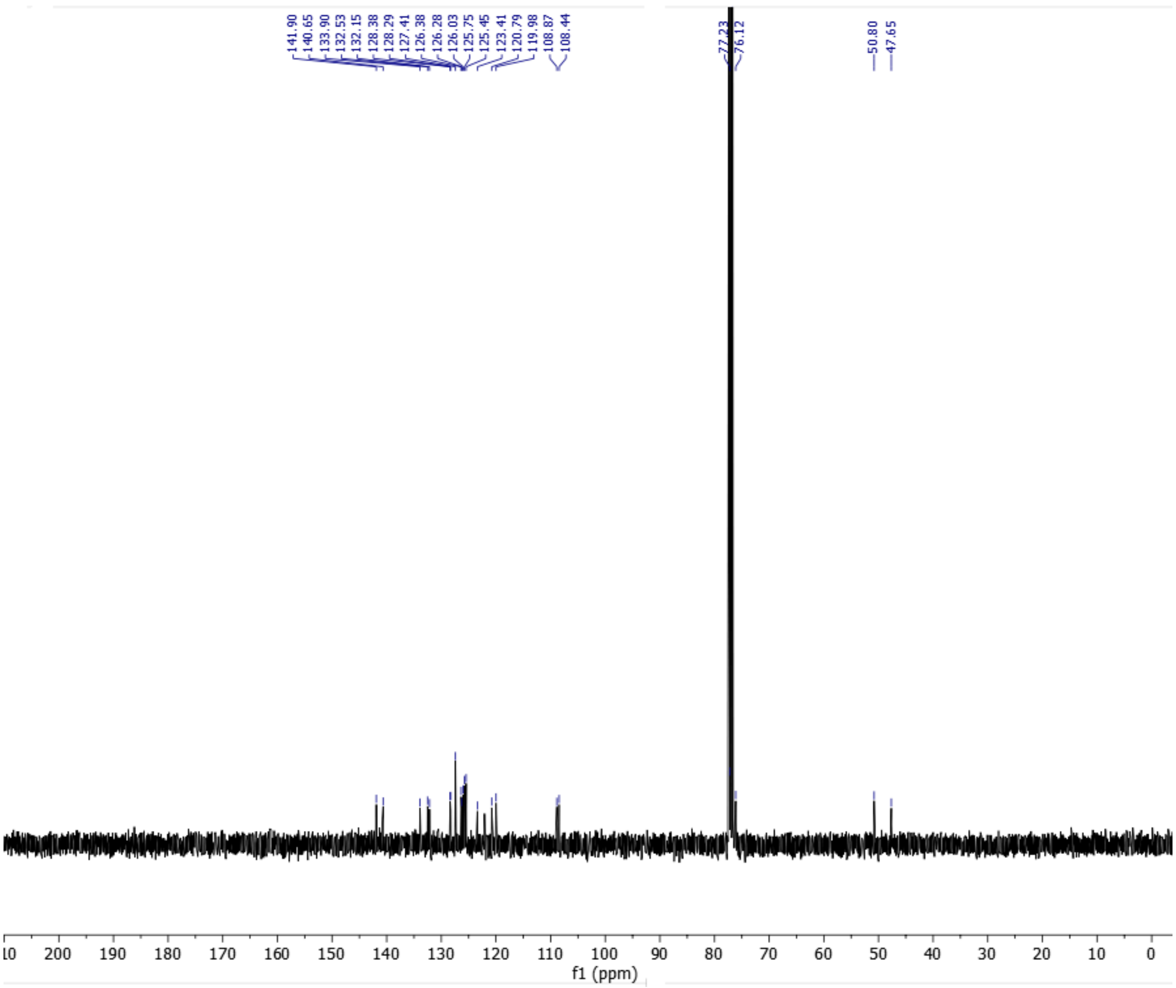

Figure S18. ^{13}C NMR of material **5.**
